# Precision medicine and parental experience: a longitudinal study of psychosocial responses to germline genomic results in pediatric oncology

**DOI:** 10.3389/fmed.2026.1890745

**Published:** 2026-08-26

**Authors:** Meaghann S. Weaver, Jessica S. Flynn, Jiaming Li, Gabrielle M. Armstrong, Lynn W. Harrison, Belinda N. Mandrell, Zachary T. Wooten, Liza-Marie Johnson, Kim E. Nichols, Katianne M. Howard Sharp

**Affiliations:** 1 The Bioethics Program, St. Jude Children’s Research Hospital, Memphis, TN, United States; 2 Department of Psychology and Biobehavioral Sciences, St. Jude Children’s Research Hospital, Memphis, TN, United States; 3 Department of Biostatistics, St. Jude Children’s Research Hospital, Memphis, TN, United States; 4 Department of Psychology, University of Mississippi, Oxford, MS, United States; 5 Department of Oncology, Division of Cancer Predisposition, St. Jude Children’s Research Hospital, Memphis, TN, United States; 6 Division of Nursing, St. Jude Children’s Research Hospital, Memphis, TN, United States

**Keywords:** family-centered care, genomic sequencing, germline genetic testing, pediatric oncology, pediatric palliative care, precision medicine, psychological outcomes

## Abstract

**Introduction:**

Precision medicine has become central to pediatric oncology, with germline genomic sequencing commonly integrated into routine care. Families must interpret complex genomic findings during emotionally vulnerable periods, generating mixed reactions ranging from clarity and relief to anxiety and uncertainty. Palliative care clinicians, genetic counselors, psychologists, social workers, and oncology providers may each contribute to supporting families as they interpret and integrate these findings over the course of a child’s cancer care and beyond. Little is known about the trajectory of parental emotional and cognitive responses after receiving germline sequencing results, limiting clinicians’ ability to anticipate support needs across the cancer care continuum. This study quantitatively examines parental emotional and cognitive responses across time following disclosure of germline sequencing results in a pediatric oncology setting.

**Methods:**

Parents (*n* = 218) self-reported sequencing-related distress, positive feelings, intrusive thoughts, certainty, and self-efficacy using validated measures at two longitudinal follow-up points after disclosure of their child’s germline test results. Outcomes were compared across germline test result types (pathogenic/likely pathogenic [P/LP], *n* = 31 [14%]; variants of uncertain significance [VUS], *n* = 86 [39%]; and negative, *n* = 101[46%]).

**Results:**

Parents of children receiving P/LP or P/LP+VUS results reported significantly higher distress yet greater positive feelings than parents receiving negative results. Notably, certainty and self-efficacy increased from Timepoint 1 (median 254 days following return of results) to Timepoint 2 (median 537 days). Intrusive thoughts did not significantly differ by genetic result type or change over time; however, the factors contributing to intrusive thoughts could not be determined from the current study.

**Discussion:**

These findings provide insight into how families adapt to germline genomic information following a pediatric cancer diagnosis. As precision medicine becomes increasingly embedded in pediatric oncology, structured follow-up and communication that address families’ evolving informational and psychosocial needs are essential to ensure care that is scientifically precise, emotionally attuned, and centered on the family experience.

## 1 Introduction

Precision medicine has reshaped the landscape of pediatric oncology through increasing use of genomic testing, including both tumor- and germline sequencing (1). Once conceptualized primarily as a research tool, genomic testing is now woven into routine childhood cancer care, guiding surveillance for individuals with cancer predisposition syndromes, informing therapeutic selection, and enabling cancer risk-reducing interventions for the patient and other family members (2). As genomic technologies become more integrated into clinical pathways, children and their families will increasingly encounter highly complex information at moments marked by vulnerability, uncertainty, and profound emotional weight (3, 4). With these genomic advances, attention has expanded beyond diagnostic and therapeutic utility to include patient and family understanding, interpretation, and adaptation to complex genetic information. Although survival outcomes for children with cancer have improved substantially over recent decades (5), many families continue to face significant illness-related uncertainty, treatment burden, relapse and secondary cancer risk, and complex medical decision-making into survivorship, all of which may be further complicated by the application of genetic risk information. Consequently, providing comprehensive pediatric oncology care throughout the illness trajectory necessitates a multidisciplinary approach that integrates psychosocial and supportive care. As genomics becomes embedded within pediatric cancer care, palliative care clinicians will increasingly need to be aware of and prepared for the ways in which genomic testing, genomic results, and their associated medical implications intersect with illness communication, uncertainty management, decision-making, and family coping. However, limited research has characterized how parents cognitively and emotionally respond to germline genomic results across result types and over time, constraining efforts to provide anticipatory, family-centered support.

When contemplating genomic testing, germline sequencing presents unique psychosocial considerations because findings may have implications for future cancer risk, long-term medical surveillance, and impacts on other family members. Pathogenic or likely pathogenic (P/LP) germline variants differ in their implications according to the gene, variant type, and clinical context, with variability in condition penetrance, age of risk onset, and available prevention or surveillance options potentially influencing psychosocial adaptation (6). While some P/LP findings may confer increased future cancer risk and inform recommendations for management (e.g., surveillance, prophylactic removal of at-risk organs), other P/LP findings may reveal the cause of the child’s cancer diagnosis without identifying current or future risk in other organs. Broad genomic testing may also identify unexpected incidental findings, such as new findings unrelated to the child’s cancer diagnosis, risk for adult-onset cancers, or variants of uncertain significance (VUS) (7). Patients may also receive multiple results, including both P/LP and VUS findings, multiple VUSs, or multiple P/LP findings with varying penetrance or clinical implications, which may further complicate emotional adaptation to genetic results.

Accordingly, genomic sequencing carries significant psychosocial implications for patients and their families (8, 9). When considering utility relevant to participation in a pediatric precision oncology program for their adolescent, parents emphasize a sense of optimism for possible treatment avenues, even while acknowledging that clinically actionable results are unlikely (10). Parents of children with cancer also describe both burdens and psychological benefits to whole-exome sequencing, such as reassurance, diminished guilt, and fulfillment of curiosity (11). Further, they note practical advantages, including anticipation of future needs and insight to help inform reproductive choices (11). Parents of children with solid tumors convey a strong drive to safeguard their child’s well-being, and within this context, they look to genetic information as a way to feel more equipped and confident when taking part in decisions about their child’s treatment and care (12). Following receipt of their child’s exome sequencing results, many parents of seriously ill children describe a sense of reassurance, with reduced feelings of guilt and anxiety, and express that consenting to genetic testing has allowed them to meet their perceived responsibilities as caregivers (13). Nevertheless, parent may differ in their preferences regarding the timing of testing and extent of information returned (14). Many parents view genetic testing as less imminently compelling compared to the immediate demands of a cancer diagnosis and the stresses of treatment (15). Careful consideration of the timing, the longitudinal psychosocial adaptation, and potential implications of involvement in such research remains important. Effectively incorporating precision medicine into pediatric cancer care therefore depends on access to psychosocial support that can adapt to families’ evolving circumstances (15).

Primarily cross-sectional and qualitative research suggests complex, heterogeneous parent experiences with pediatric oncology germline sequencing. A P/LP variant may provide a long-sought explanation for a child’s cancer, while simultaneously introducing new questions about prognosis or familial implications. Reported parental emotions associated with P/LP findings range from shock to relief (16). In pediatric oncology settings, negative emotions were described more frequently with pathogenic results as compared to VUS (17, 18). Reactions to VUS may include disappointment about not receiving an explanation for the child’s diagnosis (11, 18). VUS may also amplify ambiguity as well as misconceptions about the very meaning of the finding (18–20). Even negative results can evoke confusion and mixed emotions, including relief, disappointment, or persistent worry or wondering (11, 21). Parental emotional impact is notable as parents receiving results for their children experience higher levels of distress and uncertainty than adults receiving results for themselves, irrespective of diagnostic outcome (22). It is important to have familial supports in place for processing and adapting to pediatric germline results.

As genomics becomes embedded within pediatric cancer care, palliative care clinicians will increasingly need to be aware of and prepared for the ways in which genomic testing, genomic results, and their associated medical implications intersect with illness communication, uncertainty management, decision-making, and family coping. However, limited research has characterized how parents cognitively and emotionally respond to germline genomic results across result types and over time, constraining efforts to provide anticipatory, family-centered support. To prepare palliative care clinicians to understand and address the psychosocial needs of pediatric patients and their families, it is critical to understand how parents experience and adapt to germline genomic information (23). Although parent distress has been shown to vary across genetic test results (24), if and how parental cognitive and affective reactions to genetic test results change over time remains largely unknown (9). This gap has important implications for psychosocial support and family communication surrounding genetic result disclosure (25). The purpose of this study was to quantitatively examine parental psychosocial responses to germline sequencing results in the context of pediatric oncology. Specifically, we compare sequencing-related distress, positive feelings related to sequencing results, intrusive thoughts, certainty, and self-efficacy longitudinally across different germline result types.

Although genomic testing in pediatric oncology encompasses both somatic and germline approaches, germline sequencing presents unique psychosocial considerations because findings may have implications for future cancer risk, family members, and long-term medical surveillance. Pathogenic or likely pathogenic (P/LP) germline variants differ in their implications according to the gene, variant type, and clinical context, with variability in condition penetrance, age of risk onset, and available prevenetion or surveillance options potentially influencing psychosocial adaptation (6). Although some P/LP findings may confer increased future cancer risk and inform recommendations for cancer risk management (e.g., surveillance, prophylactic removal of at-risk organs), other P/LP findings may reveal the cause of the child’s cancer diagnosis without identifying current or future risk in other organs. Broad genomic testing may also identify unexpected incidental findings, such as new findings unrelated to the child’s cancer diagnosis, risk for adult-onset cancers, or variants of uncertain significance (VUS) (7). Patients may also receive multiple results, including both P/LP and VUS findings, multiple VUSs, or multiple P/LP findings with varying penetrance or clinical implications, which may further complicate emotional adaptation to genetic results.

## 2 Materials and methods

### 2.1 Study description and design

Data for this study were collected between September 2015 and August 2019 as part of the Genomes for Kids (G4K) research study at St. Jude Children’s Research Hospital (SJCRH) that investigated the feasibility of offering paired tumor and germline clinical genomic sequencing to prospectively identified pediatric oncology patients accepted for care at the institution (26). Parents and patients older than 13 were also invited to complete questionnaires assessing their experiences with sequencing, but only parent questionnaire data were analyzed as part of this study. This study was approved by the SJCRH Institutional Review Board (ClinicalTrials.gov identifier: NCT02530658).

### 2.2 Participants

Patients were eligible for participation in *G4K* if they were: (1) aged less than 22 years, (2) diagnosed with a benign or malignant solid [central nervous system (CNS) or non-CNS] or liquid tumor, and (3) had adequate tumor tissue and/or germline sample available. Exclusion criteria included: (1) history of hematopoietic stem cell transplantation or (2) unwillingness of patient or legal guardian (“parents”) to provide written informed consent. To participate in questionnaires, parents were also required to be fluent in English and agree to the return of germline results.

### 2.3 Procedures

Trained staff approached eligible patient families at least 1 month after arriving at SJCRH to consider study participation; after patients (age ≥ 18 years) or parents provided informed consent, germline sequencing was completed at SJCRH. At informed consent, parents completed a demographic form, querying patient age, sex, race, and ethnicity, as well as parent age, sex, relationship status, educational attainment, and annual family income.

Genetic counselors disclosed germline results to families who agreed to learn results. Results included P/LP (one or more P/LP cancer-predisposing germline variants), VUS (one or more VUS), P/LP+VUS (both at least one P/LP variant and at least one VUS; analyzed as a distinct result category), or negative (no cancer-predisposing germline variants were identified) (26). Subsequently, parents completed paper-and-pencil questionnaires in person at two time points after disclosure (T1: ≥5 weeks; T2: ≥19 weeks). Timing of questionnaire completion remained flexible to allow for completion in person when their child was at SJCRH for care. Questionnaires were not sought from parents whose child was deceased. Parents and patients did not receive compensation for participation. Medical chart review was performed by trained study staff to collect patient clinical features, such as tumor type and relapse status, and missing family demographics.

### 2.4 Measures

#### 2.4.1 Impact of Genetic Testing

The Impact of Genetic Testing (IGT) is a 16-item self-report measure developed to assess the psychological impact of receiving genetic susceptibility information and was originally developed in the context of Alzheimer’s disease testing (27). For the present study, item wording was modified to reflect parents’ distress and positive impact in relation to their child’s cancer risk and sequencing results. Items were rated on a 4-point Likert-type scale (0 = never, 1 = rarely, 3 = sometimes, and 5 = often) and summed to create two subscale composites, with higher scores indicating greater levels of psychological impact. The IGT was administered to all parents regardless of genetic test result. For the current study, only the two items focusing on positive emotions (i.e., feeling relieved or happy) were summed from the positive impact scale, with scores ranging from 0 to 10 at both timepoints. These two items were selected to reflect positive genomic sequencing feelings specifically rather than broad positive experiences (which incorporated positive family communication experiences beyond the scope of the research question). Positive feelings demonstrated excellent internal consistency (α = 0.92 and 0.93). In the present study, the distress subscale ranged from 0 to 44 at T1 and 0–46 at T2, with acceptable-to-good internal consistency, respectively (α = 0.79 and 0.81).

#### 2.4.2 Psychological Adaptation to Genetic Information

The Psychological Adaptation to Genetic Information Scale (PAGIS) is a validated 26-item measure developed to assess how individuals psychologically adapt to learning genetic information (28). The PAGIS includes five subscales: non-intrusive thoughts, social support, self-worth, certainty, and self-efficacy. For the present study, parents of children with a P/LP result and/or VUS completed the non-intrusive thoughts, certainty, and self-efficacy subscales to report their own adaptation to their child’s genomic sequencing. These subscales of the PAGIS were selected to reflect hypothesized affective and cognitive parental outcomes of sequencing. Parents receiving negative sequencing results did not complete the PAGIS; therefore, analyses involving intrusive thoughts, certainty, and self-efficacy were restricted to the P/LP, VUS, and P/LP+VUS groups. The 15 items were rated on a 5-point Likert-type scale (1 = strongly disagree to 5 = strongly agree), with higher scores indicating better psychological adaptation. However, items were reverse coded for the non-intrusive thoughts subscale for ease of interpretation to reflect intrusive thoughts, with lower scores reflecting fewer intrusive thoughts and therefore lower scores on this subscale indicate better psychological adaptation. Subscale scores were calculated using the mean of items in each subscale and could range from 1 to 5. The PAGIS has demonstrated sound psychometric properties (28). In the present study, internal consistency was acceptable to good (αs ranged from 0.77 [self-efficacy, T2] to 0.89 [self-efficacy, T1]).

### 2.5 Analyses

Data were analyzed using separate mixed-effect regression models with a random intercept at the participant level for each outcome: distress, positive feelings, intrusive thoughts, certainty, and self-efficacy. Each model included genetic test result (P/LP, VUS, P/LP+VUS, or negative), timepoint (T1 vs. T2), and the interaction between timepoint and genetic test result to assess whether outcome changes over time depended on genetic test result. T1 served as the reference for timepoint. Negative results served as the reference group for models examining distress and positive feelings. Given that the PAGIS was administered only to parents of children with P/LP and/or VUS, the VUS-only group served as the reference category for models examining intrusive thoughts, certainty, and self-efficacy. To account for variability in questionnaire timing, days since result disclosure was included in all models as a covariate. The adjusted Generalized Variance Inflation Factor (GVIF) was used to assess the potential multicollinearity between covariates. Models were fit using restricted maximum likelihood estimation, which allows incomplete longitudinal data to be incorporated. Hence, observations with missing model variables were excluded only at the affected time point, while participants with data from only T1 or T2 were retained in the models. To account for potential clustering of multiple parents within the same child/family, a sensitivity analysis was conducted using a nested mixed-effects model with parents nested within child, retaining the same fixed-effects structure as the primary analysis. As this was a retrospective study, sample size was not predetermined by *a priori* power calculation. *Post hoc* power was estimated via simulation (1,000 iterations) for each coefficient within each outcome model based on observed effect sizes (Supplementary Table 1). The unstandardized effect sizes are reported for consistency to be interpreted directly on each outcome’s original measurement scale. The five outcomes were prespecified and hypothesis-driven, each reflecting a distinct psychological construct assessed independently. Each outcome was analyzed in a separate model for prespecified genetic groups and model-based comparisons; therefore, no adjustment for multiple comparisons was applied.

## 3 Results

### 3.1 Descriptive analyses

Of the 363 families approached, 310 patients consented (85.4%) to the larger G4K study, with parents of 196 children (63.2%) agreeing to questionnaire participation. Notably, some families were not approached for surveys due to linguistic limitations in the inclusion criteria and some families consented to surveys but did not complete the surveys due to bereavement. Post-disclosure questionnaires were completed by 218 parents (155 maternal caregivers, 63 paternal caregivers) of 170 children (86.7%). Participants were assessed at a median of 264 days (range: 37–840) following return of results at Timepoint 1 and 537 days (range: 139–1090) at Timepoint 2 following return of results. The corresponding mean (SD) times since ROR were 294 (178) and 525 (203) days, respectively. Most parents completed both T1 and T2 questionnaires (*n* = 153; 70.2%); however, 41 parents completed only T1 (18.8%) and 24 parents completed only T2 (11.0%). Thus, analyses included data from 194 parents at T1 to 177 parents at T2.

As previously reported, families of Black children were more likely than families of White Hispanic or Non-Hispanic children to decline participation in the larger *G4K* study (29). Several significant differences were also found between parents who did and did not complete T1 and T2, with fewer paternal caregivers than maternal caregivers, χ^2^(1) = 23.1, *p* < 0.001, more parents of children with liquid tumor, χ^2^(2) = 6.6, *p* = 0.04, completing T1, and parents reporting annual income < $60,000 less likely to complete T2, χ^2^(1) = 6.2, *p* = 0.01. Parents with complete data did not significantly differ from those with missing data on other key variables (i.e., germline result, relapse status, ethnicity/race, gender, marital status, formal education of parent, or age of parent or patient age). Demographic and clinical characteristics for participants completing questionnaires are reported in Table 1.

**TABLE 1:** Demographic and clinical characteristics, stratified by germline sequencing result.

		Germline result		
Children’s demographic and clinical variables	Overall	Negative	P/LP	VUS
No.	170	79	21	70
Age (at consent), years				
Mean (SD)	7.23 (5.4)	7.7 (5.6)	7.4 (6.3)	6.7 (4.8)
Range	0.2–21.7	0.4–21.7	0.3–17.8	0.2–17.9
Sex, No. (%)				
Male	84 (49.4)	42 (53.2)	11 (52.4)	31 (44.3)
Female	86 (50.6)	37 (46.8)	10 (47.6)	39 (55.7)
Race, No. (%)				
White	136 (80.0)	67 (84.8)	15 (71.4)	54 (77.1)
Other races^a^	34 (20.0)	12 (15.2)	6 (28.6)	16 (22.9)
Ethnicity, No. (%)				
Non-Hispanic	154 (90.6)	76 (96.2)	19 (90.5)	59 (84.3)
Hispanic	16 (9.4)	3 (3.8)	2 (9.5)	11 (15.7)
Tumor type, No. (%)				
CNS	50 (29.4)	22 (27.8)	7 (33.3)	21 (30.0)
Liquid	72 (42.4)	36 (45.6)	4 (19.1)	32 (45.7)
Solid	48 (28.2)	21 (26.6)	10 (47.6)	17 (24.3)
Relapse, No. (%)				
Newly diagnosed	147 (86.5)	71 (89.9)	16 (76.2)	60 (85.7)
Relapsed, refractory, or secondary cancer	23 (13.5)	8 (10.1)	5 (23.8)	10 (14.3)
		**Germline result**		
**Parents’ demographic variables**	**Overall**	**Negative**	**P/LP**	**VUS**
No.	218	101	31	86
Age, years				
Mean (SD)	36.3 (8.1)	37.4 (8.1)	35.6 (7.3)	35.2 (8.3)
Range	17.6–65.6	20.1–65.6	21.2–51.9	17.6–55.1
Sex, No. (%)				
Male	63 (28.9)	30 (29.7)	11 (35.5)	22 (25.6)
Female	155 (71.1)	71 (70.3)	20 (64.5)	64 (74.4)
Relationship status,b No. (%)				
Single	42 (19.3)	17 (16.8)	6 (19.4)	19 (22.1)
Partnered	158 (72.5)	76 (75.2)	23 (74.2)	59 (68.6)
Education,b No. (%)				
<College graduate	98 (45.0)	43 (42.6)	16 (51.6)	39 (45.3)
≥College graduate	96 (44.0)	49 (48.5)	7 (22.6)	40 (46.5)
Family income,b USD, No. (%)				
<$60,000	94 (43.1)	43 (42.6)	10 (32.3)	41 (47.7)
≥$60,000	87 (39.9)	46 (45.5)	9 (29.0)	32 (37.2)
T1 data collected				
No. (%)	194 (89.0)	91 (90.0)	25 (80.6)	78 (90.7)
Time since disclosure at T1, weeks				
Mean (SD)	42.1 (25.4)	45.1 (24.5)	31.3 (19.0)	42.2 (27.3)
Range	5.0–120.0	7.0–120.0	6.0–57.0	5.0–116.0
T2 data collected				
No. (%)	177 (81.2)	76 (75.2)	27 (87.1)	74 (86.0)
Time since disclosure at T2, weeks				
Mean (SD)	75.3 (28.4)	78.3 (28.0)	68.6 (23.7)	74.7 (30.1)
Range	19.0–156.0	19.0–155.0	33.0–99.0	25.0–156.0

P/LP, pathogenic/likely pathogenic; VUS, variant of uncertain significance; No., number; SD, standard deviation; CNS, central nervous system; USD, US dollars.

*^a^*Other races = races other than White.

*^b^*Percentages may not add up to 100% due to missing data.

Parental group sizes included negative (*n* = 101, 46%), VUS (*n* = 86, 39%), and P/LP (*n* = 31, 14%) result categories. Representative pathogenic and likely pathogenic findings included variants in established cancer predisposition genes such as TP53, RB1, APC, PMS2, and BRCA2. Variants of uncertain significance were identified in a range of cancer-associated genes in which available evidence at the time of reporting was insufficient to determine pathogenicity. As previously described by the study team (26), examples included missense variants in genes such as ATM, CHEK2, PALB2, and other hereditary cancer susceptibility genes.

Across all genetic result groups, observed mean (SD) distress scores were 8.81 (7.87) at Timepoint 1 (T1) and 8.67 (7.99) at Timepoint 2 (T2); observed mean (SD) positive feelings scores were 3.07 (3.78) at Timepoint 1 and 3.07 (3.80) at Timepoint 2.

### 3.2 Mixed effects regression models

A summary of all models can be found in Table 2. A summary of results from the clustering-adjusted sensitivity analyses can be found in Supplementary Table 3. Results were consistent with the primary analysis; the only notable difference was that the Time × P/LP+VUS interaction for distress changed from statistically significant (*p* = 0.041) to borderline significance (*p* = 0.063) in the clustering-adjusted model.

**TABLE 2:** Mixed effects regression models.

Outcome	Estimate	95% CI	*P*
Distress			
Timepoint	−0.70	−2.61, 1.21	0.469
P/LP result	6.94	2.36, 11.53	**0.003**
VUS result	1.37	−1.01, 3.74	0.258
P/LP+VUS result	8.70	4.08, 13.32	**<0.001**
Days since result disclosure	0.001	−0.004, 0.006	0.727
Timepoint × P/LP result	0.19	−3.97, 4.36	0.927
Timepoint × VUS result	0.16	−2.05, 2.37	0.886
Timepoint × P/LP+VUS result	−4.55	−8.91, −0.20	**0.041**
Positive feelings			
Timepoint	−0.32	−1.32, 0.67	0.525
P/LP result	2.82	0.74, 4.90	**0.008**
VUS result	1.63	0.52, 2.74	**0.004**
P/LP+VUS result	3.67	1.50, 5.84	**0.001**
Days since result disclosure	0.002	−0.000, 0.004	0.055
Timepoint × P/LP result	1.12	−1.08, 3.32	0.316
Timepoint × VUS result	−0.19	−1.38, 1.00	0.754
Timepoint × P/LP+VUS result	0.31	−2.02, 2.65	0.791
Intrusive thoughts			
Timepoint	−0.10	−0.34, 0.15	0.429
P/LP result	0.23	−0.22, 0.69	0.316
P/LP+VUS result	0.12	−0.36, 0.60	0.620
Days since result disclosure	−0.000	−0.001, 0.001	0.876
Timepoint × P/LP result	0.18	−0.33, 0.68	0.483
Timepoint × P/LP+VUS result	0.15	−0.40, 0.70	0.588
Certainty			
Timepoint	0.31	0.09, 0.53	**0.006**
P/LP result	0.32	−0.12, 0.76	0.151
P/LP+VUS result	0.48	0.03, 0.94	**0.038**
Days since result disclosure	−0.001	−0.002, −0.0003	**0.004**
Timepoint × P/LP result	−0.15	−0.60, 0.30	0.502
Timepoint × P/LP+VUS result	0.04	−0.46, 0.53	0.887
Self-efficacy			
Timepoint	0.36	0.08, 0.63	**0.011**
P/LP result	0.26	−0.21, 0.73	0.276
P/LP+VUS result	−0.19	−0.67, 0.30	0.448
Days since result disclosure	−0.001	−0.002, −0.0004	**0.001**
Timepoint × P/LP result	−0.41	−1.01, 0.18	0.173
Timepoint × P/LP+VUS result	0.31	−0.34, 0.96	0.343

Bolding reflects statistical significance, *p* < 0.05; P/LP, pathogenic/likely pathogenic; VUS, variant of uncertain significance. Negative result served as the reference group for distress and positive feelings; VUS served as the reference group for intrusive thoughts, certainty, and self-efficacy. T1 used as the reference group for timepoint.

#### 3.2.1 Distress

There was a significant overall effect of genetic test result on sequencing-related distress (Type III test *p* < 0.001). Compared with parents of children receiving negative results, parents of children receiving P/LP-only or P/LP+VUS results endorsed significantly higher distress at T1 (P/LP only: *b* = 6.94, 95% CI: 2.36–11.53, *p* = 0.003, P/LP+VUS: *b* = 8.70, 95% CI: 4.08–13.32, *p* < 0.001). At T1, estimated distress was 7.75 (95% CI: 6.07–9.43) for the negative group, 14.70 (95% CI: 10.34–19.05) for the P/LP-only group, and 16.45 (95% CI:12.07–20.83) for the P/LP+VUS group (Supplementary Table 2). Parents of children with VUS-only results did not significantly differ in distress from those receiving negative results. Across all participants, distress scores did not significantly change from T1 to T2, indicating no overall effect of time on distress. The overall Timepoint × Genetic Result interaction was not significant (Type III test, *p* = 0.19), indicating no strong evidence that group differences changed over time. Although the individual P/LP+VUS × Time contrast reached nominal significance (*b* = −4.55, 95% CI: −8.91 to −0.20, *p* = 0.041), this pattern did not persist in the sensitivity analysis accounting for within-family clustering (*p* = 0.063). Therefore, this finding should be interpreted with caution given the non-significant omnibus interaction test.

#### 3.2.2 Positive feelings

The overall effect of genetic test result was significant on sequencing-related positive feelings (Type III test: *p* < 0.001), indicating significant differences in positive sequencing-related emotions across genetic results (Table 2). Compared with parents of children receiving a negative result, T1 positive emotions scores were significantly higher among those receiving P/LP-only, VUS-only, and P/LP+VUS results (P/LP only: *b* = 2.82, 95% CI: 0.74–4.90, *p* = 0.008, VUS only: *b* = 1.63, 95% CI: 0.52–2.74, *p* = 0.004, P/LP+VUS: *b* = 3.67, 95% CI: 1.50–5.84, *p* = 0.001). At T1, estimated positive feelings scores were 2.11 (95% CI: 1.32–2.89) for negative group, 4.93 (95% CI: 2.96–6.90) for the P/LP-only group, 3.74 (95% CI: 2.90–4.58) for the VUS-only group, and 5.78 (95% CI: 3.72–7.83) for the P/LP+VUS group (Supplementary Table 2). Positive feelings scores did not significantly change over time or vary over time by genetic results.

#### 3.2.3 Intrusive thoughts

Intrusive thoughts did not significantly differ by genetic result, change over time, or vary over time by genetic result (Table 2).

#### 3.2.4 Certainty

There was a significant overall effect of genetic test result on certainty (Type III test: *p* = 0.03). Specifically, at T1 parents of children with P/LP+VUS endorsed significantly higher certainty compared to the VUS only group (*b* = 0.48, 95% CI: 0.03–0.94, *p* = 0.038). At T1, estimated certainty scores were 3.33 (95% CI: 3.16–3.51) for VUS only group, and 3.82 (95% CI: 3.38–4.25) for P/LP+VUS group (Supplementary Table 2). Certainty was also significantly associated with timepoint (Type III test: *p* = 0.040), with significantly higher certainty endorsed at T2 relative to T1 for the VUS only group (*b* = 0.31, 95% CI: 0.09–0.53, *p* = 0.006). At T1, estimated certainty scores were 3.33 (95% CI: 3.16–3.51) for VUS only group, and the scores were 3.65 (95% CI: 3.45–3.84) for VUS only group at T2 (Supplementary Table 2); however, timepoint did not significantly interact with genetic test result. Greater days since result disclosure was associated with lower score in certainty (*b* = −0.001, 95% CI: −0.002 to −0.0003, *p* = 0.004). Days-since-disclosure variable captured variability in assessment timing within each study timepoint.

#### 3.2.5 Self-efficacy

There was a significant overall effect of timepoint on self-efficacy (Type III test *p* = 0.049), with self-efficacy significantly increasing from T1 to T2 for the VUS only group (*b* = 0.36, 95% CI: 0.08–0.63, *p* = 0.011). At T1,the estimated mean of self-efficacy was 3.41 (95% CI: 3.22–3.60) for VUS-only group, and self-efficacy was 3.76 (95% CI: 3.56–3.97) for VUS-only group at T2 (Supplementary Table 2). In contrast, there was no significant effect of genetic test result and no significant interaction between time and genetic test result. Greater time since result disclosure was associated with lower self-efficacy (*b* = −0.001, 95% CI: −0.002 to −0.0004, *p* = 0.001). This finding reflects within-timepoint variation in questionnaire timing rather than the overall longitudinal change observed between T1 and T2.

## 4 Discussion

This study examined parental psychosocial responses following return of germline sequencing results for pediatric oncology patients participating in a research study of clinical genomic sequencing of paired tumor and normal tissues. Several important patterns emerged that help clarify how families may process genetic information in the months following disclosure. Specifically, the study identified meaningful variation in genetic test-related distress, positive feelings after receiving results, certainty, and self-efficacy across genetic result types and longitudinal timepoints.

### 4.1 Distress responses to genetic test results

Distress levels varied significantly by genetic test result. Parents receiving P/LP results (either alone or in combination with a VUS) reported significantly higher distress than parents receiving negative results. In contrast, distress among parents receiving VUS-only results did not differ from those receiving negative results. These findings are consistent with our previously published research (18). These findings suggest that the presence of identified genetic risk or potential changes to medical management associated with pathogenic findings, rather than uncertainty from a VUS, may be the primary driver of elevated distress. Importantly, the degree of clinical actionability likely varied across participants, and not all pathogenic findings necessarily resulted in changes to medical management; thus, future research should examine how parent distress may vary according to the actionability of the gene variant.

Although parents receiving P/LP+VUS results demonstrated the highest initial distress, descriptive trends suggested some reduction in distress over time for this group consistent with the broader literature (30–32); however, the overall time by result interaction was not statistically significant, so this pattern should be interpreted with caution. Evidence from adult genetic testing studies suggests reductions in distress and anxiety over time (30, 31). This temporal decline aligns with models of adaptive coping (32), in which initial emotional reactions to high-impact health information gradually attenuate as families integrate the information into clinical care and daily life.

These findings are broadly consistent with larger longitudinal studies of genomic result disclosure. In a meta-analysis of seven Clinical Sequencing Exploratory Research (CSER) Consortium studies involving both adult and pediatric populations, Robinson et al. reported generally modest psychological effects following exome and genome sequencing, with little evidence of sustained clinically significant distress among most participants. Similar studies have suggested that while distress may increase following the receipt of medically significant findings, many individuals demonstrate adaptation over time. Our findings extend this literature by suggesting that parents receiving both a P/LP result and a VUS may experience a distinct trajectory of distress, although this observation warrants cautious interpretation given the small subgroup size and non-significant omnibus interaction test (33).

### 4.2 Positive feelings after receiving genetic results

Positive feelings were higher among parents receiving all three non-negative result types (P/LP-only, VUS-only, and P/LP+VUS) compared with those receiving negative results. These findings mirror extensive qualitative literature describing “mixed emotions” among families receiving genetic diagnoses in the context of childhood cancer including relief, validation, and increased clarity about cancer etiology and future risks, alongside worry or sadness (9, 18). Prior work has emphasized that obtaining an explanation for a child’s cancer can generate meaning and provide direction for surveillance or prevention (34), even when pathogenic findings also introduce concern. Our quantitative results corroborate these qualitative themes, demonstrating that increased distress does not preclude simultaneous emergence of positive affective responses. Conversely, experiences of relief, reassurance, and reduced uncertainty following genetic results do not exclude the simultaneous presence of ongoing worry or grief, reflecting the coexistence of positive and negative emotional responses within parental coping processes. Notably, interpretation of the positive feelings outcome should be made within the context of our narrow measurement of positive feelings, which reflects relief and happiness following sequencing disclosure. These findings may not comprehensively reflect the broader construct of positive impact, including dimensions such as empowerment, reduced guilt, future planning, family communication, or improved understanding of genetic risk.

The coexistence of positive and negative emotions observed in our cohort is consistent with the broader pediatric genomic sequencing literature. In the BabySeq randomized clinical trial, parents frequently reported perceived benefits of genomic sequencing despite the potential for uncertainty or worry associated with the information returned (35). Likewise, longitudinal studies of genomic diagnosis in infants have demonstrated that parents may simultaneously experience relief, validation, concern, and uncertainty as they integrate genetic information into their understanding of their child’s condition (36, 37). These findings support the concept that parental adaptation to genomic results is multidimensional rather than uniformly positive or negative.

The concurrent presence of elevated distress and heightened positive reactions among individuals receiving P/LP results relative to negative results may indicate that pathogenic findings exert a greater overall psychosocial impact, amplifying both the challenges and the perceived significance of the testing experience. Genetic counselors and interdisciplinary colleagues can support families in acknowledging and integrating a “both/and” framework (holding hope, relief, or reassurance alongside fear, worry, or grief) rather than feeling compelled toward an “either/or” interpretation when processing complex genetic news. This finding reminds us that genetic test results benefit from interpretation and longitudinal processing in the context of a relational care model (38).

### 4.3 Intrusive thoughts

Unlike distress and positive feelings, intrusive thoughts did not differ by genetic result group, change over time, or vary longitudinally by result type. Intrusive thought levels were generally low, suggesting that while genetic testing elicited meaningful emotional responses, it did not lead to persistent cognitive intrusion for most parents. One possible explanation is that intrusive thoughts may be triggered by specific events, such as the emergence of symptoms in at-risk organs or anticipation of future medical surveillance, rather than reflecting a sustained response to genetic test disclosure. Alternatively, our measure may not have captured other forms of rumination or intrusive thinking relevant to adaptation following genetic testing.

### 4 4 Certainty about understanding the genetic result

Certainty increased significantly from T1 to T2, suggesting that parents generally felt more confident in their understanding of the genetic information over time. Parents of children with P/LP+VUS results reported significantly higher certainty relative to those receiving VUS-only results. This may indicate that clinically meaningful findings, some of which may carry actionable implications, enhance clarity, whereas VUS-only results, characterized by inherent ambiguity, may impede parents’ confidence in their understanding. One additional consideration is that individuals receiving P/LP results are more likely to have follow-up appointments with a genetic counselor, which may have enhanced both their understanding of the results and their confidence in that understanding. As families with negative or VUS-only results may have limited ongoing contact with genetic counseling services, additional interdisciplinary team members may be well positioned to reiterate and contextualize information regarding the implications, or lack thereof, associated with these results.

Increasing certainty over time has also been observed in other genomic sequencing cohorts. Studies from the BabySeq Project and related pediatric sequencing programs suggest that parents often become more comfortable with and confident in their understanding of genomic information as opportunities for follow-up discussion and clinical application emerge (35). Together, these findings support an ongoing process of adaptation extending beyond result disclosure conversations.

### 4.5 Self-efficacy

Self-efficacy increased from T1 to T2, demonstrating that parents also generally felt more capable of managing or acting upon genetic information over time. Unlike other outcomes, self-efficacy did not differ across genetic result groups. This suggests that parental confidence in their ability to navigate genetic information may be influenced more by factors such as timing, communication, or support processes than by the specific result type. Over time, increases in both certainty and self-efficacy suggest a process of adaptive integration whereby parents may progressively incorporate genetic information into their understanding and management of their child’s diagnosis. The observed increase in self-efficacy over time is similarly consistent with longitudinal models of genetic adaptation, which propose that individuals progressively integrate genetic information into their understanding of health, illness, and future planning (33, 36). Increased confidence over time has been reported in studies examining families’ responses to genomic information across pediatric settings (33, 36), suggesting that adaptation continues beyond the initial disclosure encounter.

Although average certainty and self-efficacy increased from T1 to T2, greater days since disclosure were associated with lower certainty and self-efficacy in the regression models. These findings are not necessarily contradictory. The T1 versus T2 comparisons reflect average differences between two assessment periods, whereas the days since disclosure covariate captures variation in assessment timing within those periods. Because questionnaire completion was intentionally flexible and linked with patients’ existing medical follow-up appointments, these findings may also reflect heterogeneity in adaptation trajectories among families with varying intervals between medical follow-up visits.

### 4.6 Limitations

Several characteristics of the study sample and setting may limit the generalizability of these findings. This study was conducted at a single institution with a strong clinical and cultural orientation toward genetic testing, which may differ from settings where genetic testing is less routinely integrated into care. In addition, genomic sequencing was conducted within a research protocol rather than as part of standard clinical care, potentially influencing participant expectations, support structures, and interpretation of results. Participation required English fluency and willingness to receive germline sequencing results, bereaved families were not surveyed, and families of Black children were more likely to decline participation in the larger G4K study. Differential attrition also may affect representativeness, as paternal caregivers were more likely to have missing data at T1 and parents with lower family income were more likely to have missing data at T2. Consequently, the experiences represented in this cohort may not fully reflect those of non-English-speaking families, bereaved parents, racial and ethnic minority groups, fathers, or families with fewer socioeconomic resources. These populations may experience genomic testing, uncertainty, communication, and psychosocial adaptation differently, underscoring the need for future studies in more diverse and representative samples.

Several methodological limitations should also be considered when interpreting these findings. Although T1 and T2 were analyzed as discrete assessment periods, participants within each group did not complete questionnaires at uniform intervals following result disclosure. While days since disclosure was included as a covariate, substantial variability within assessment windows may have influenced estimates of adaptation and contributed to differences observed between timepoint effects and disclosure-timing effects. In addition, genomic result groups were unequal in size, with relatively fewer participants in the P/LP-only and P/LP+VUS categories, which may have reduced power to detect modest between-group differences. Measurement limitations should also be noted. The positive feelings outcome was derived from two emotion-focused IGT items (relief and happiness) and therefore represents a narrower construct than the full subscale. Furthermore, intrusive thoughts, certainty, and self-efficacy were assessed only among parents receiving P/LP and/or VUS results, precluding comparisons with families who received negative results. *Post hoc* power for the five outcomes are provided in Supplementary Table 1. We note that *post hoc* power calculated from observed effect sizes is mathematically related to the observed *p*-value and should be interpreted with caution. Despite these limitations, this study provides quantitative insight into parents’ affective and cognitive adaptation to their child’s genetic test results across time and result types. These patterns highlight opportunities for palliative care and interdisciplinary clinicians to provide ongoing support that is responsive to families’ evolving informational and psychosocial needs following genomic testing.

## 5 Conclusion

Overall, the findings of this study highlight a nuanced emotional and experiential landscape for parents receiving pediatric germline sequencing results. Pathogenic findings may generate higher distress, yet they may also coincide with higher positive feelings. This study suggests that clinically meaningful or explanatory results evoke complex, multi-dimensional responses. Over time, both certainty and self-efficacy appear to increase, suggesting adaptive integration of genetic information. Despite ranges in scores across measures, mean trends indicate that, on average, this pattern of adaption holds true. These findings suggest that some parents move toward adaptive integration of genetic information over time. Together, the results illuminate the importance of structured follow-up, interdisciplinary communication, and supportive counseling to help families interpret and integrate genetic findings over time. Additional research is warranted to identify key facilitators of adaptation, including the potential role of palliative care services, supportive interventions, and interdisciplinary partnerships that may enhance families’ capacity to integrate and cope with genetic information over time.

## Data Availability

The raw data supporting the conclusions of this article will be made available by the authors, without undue reservation.
