# Supplementary material for: Precision medicine and parental experience: a longitudinal study of psychosocial responses to germline genomic results in pediatric oncology
Source: Front Med (Lausanne). 2026 Aug 26;13:1890745. doi: 10.3389/fmed.2026.1890745 (PMC13553464; doi:10.3389/fmed.2026.1890745)
Supplement: Supplementary file 1 [file Table_1.docx]

Supplementary Material

# 1 Supplementary Data

Table S1. Post hoc power analysis for the outcome models.

| Outcome | Effect Size | Alpha | power (%) | Lower 95% CI | Upper 95% CI |
| --- | --- | --- | --- | --- | --- |
| **Distress** |  |  |  |  |  |
| Timepoint | -0.70 | 0.05 | 11.2 | 9.31 | 13.32 |
| P/LP only result | 6.94 | 0.05 | 85.3 | 82.95 | 87.44 |
| VUS result | 1.37 | 0.05 | 19.9 | 17.47 | 22.51 |
| P/LP + VUS result | 8.70 | 0.05 | 96.1 | 94.71 | 97.21 |
| Days since result disclosure | 0.001 | 0.05 | 5.3 | 3.99 | 6.88 |
| Timepoint*P/LP result | 0.19 | 0.05 | 4.5 | 3.30 | 5.98 |
| Timepoint*VUS result | 0.16 | 0.05 | 3.6 | 2.53 | 4.95 |
| Timepoint*P/LP + VUS result | -4.55 | 0.05 | 52.8 | 49.65 | 55.93 |
| **Positive Feelings** |  |  |  |  |  |
| Timepoint | -0.32 | 0.05 | 11.5 | 9.59 | 13.64 |
| P/LP only result | 2.82 | 0.05 | 78.6 | 75.93 | 81.10 |
| VUS result | 1.63 | 0.05 | 82.1 | 79.58 | 84.43 |
| P/LP + VUS result | 3.67 | 0.05 | 92 | 90.14 | 93.61 |
| Days since result disclosure | 0.002 | 0.05 | 50.6 | 47.45 | 53.74 |
| Timepoint*P/LP result | 1.12 | 0.05 | 16.5 | 14.25 | 18.95 |
| Timepoint*VUS result | -0.19 | 0.05 | 7 | 5.50 | 8.76 |
| Timepoint*P/LP + VUS result | 0.31 | 0.05 | 6.2 | 4.79 | 7.88 |
| **Intrusive Thoughts** |  |  |  |  |  |
| Timepoint | -0.10 | 0.05 | 11.5 | 9.59 | 13.64 |
| P/LP result | 0.23 | 0.05 | 18.2 | 15.85 | 20.73 |
| P/LP + VUS result | 0.12 | 0.05 | 8.5 | 6.85 | 10.40 |
| Days since result disclosure | -0.000 | 0.05 | 5.1 | 3.82 | 6.65 |
| Timepoint*P/LP result | 0.18 | 0.05 | 11.8 | 9.87 | 13.96 |
| Timepoint*P/LP + VUS result | 0.15 | 0.05 | 9.1 | 7.39 | 11.06 |
| **Certainty** |  |  |  |  |  |
| Timepoint | 0.31 | 0.05 | 79.1 | 76.45 | 81.58 |
| P/LP result | 0.32 | 0.05 | 31.9 | 29.02 | 34.89 |
| P/LP + VUS result | 0.48 | 0.05 | 56.1 | 52.96 | 59.20 |
| Days since result disclosure | -0.001 | 0.05 | 83.8 | 81.37 | 86.03 |
| Timepoint*P/LP result | -0.15 | 0.05 | 9.8 | 8.03 | 11.81 |
| Timepoint*P/LP + VUS result | 0.04 | 0.05 | 6.4 | 4.96 | 8.10 |
| **Self-Efficacy** |  |  |  |  |  |
| Timepoint | 0.36 | 0.05 | 74.1 | 71.27 | 76.79 |
| P/LP result | 0.26 | 0.05 | 18.8 | 16.42 | 21.36 |
| P/LP + VUS result | -0.19 | 0.05 | 14.8 | 12.66 | 17.15 |
| Days since result disclosure | -0.001 | 0.05 | 93.8 | 92.12 | 95.21 |
| Timepoint*P/LP result | -0.41 | 0.05 | 29.7 | 26.88 | 32.64 |
| Timepoint*P/LP + VUS result | 0.31 | 0.05 | 15.8 | 13.59 | 18.21 |

Table S2. Estimated marginal means by timepoint and genetic result

| Outcome | time | GLresult | Estimated | 95% CI |
| --- | --- | --- | --- | --- |
|  |  |  | Mean |  |
| Distress |  |  |  |  |
|  | Follow-up 1 | Negative | 7.75 | 6.07, 9.43 |
|  | Follow-up 2 | Negative | 7.05 | 5.20, 8.90 |
|  | Follow-up 1 | P/LP only | 14.70 | 10.34, 19.05 |
|  | Follow-up 2 | P/LP only | 14.19 | 10.04, 18.34 |
|  | Follow-up 1 | VUS only | 9.12 | 7.32, 10.92 |
|  | Follow-up 2 | VUS only | 8.58 | 6.74, 10.42 |
|  | Follow-up 1 | P/LP + VUS | 16.45 | 12.07, 20.83 |
|  | Follow-up 2 | P/LP + VUS | 11.20 | 6.73, 15.67 |
| Positive Feelings |  |  |  |  |
|  | Follow-up 1 | Negative | 2.11 | 1.32, 2.89 |
|  | Follow-up 2 | Negative | 1.79 | 0.90, 2.67 |
|  | Follow-up 1 | P/LP only | 4.93 | 2.96, 6.90 |
|  | Follow-up 2 | P/LP only | 5.73 | 3.87, 7.59 |
|  | Follow-up 1 | VUS only | 3.74 | 2.90, 4.58 |
|  | Follow-up 2 | VUS only | 3.23 | 2.35, 4.11 |
|  | Follow-up 1 | P/LP + VUS | 5.78 | 3.72, 7.83 |
|  | Follow-up 2 | P/LP + VUS | 5.77 | 3.66, 7.88 |
| Intrusive Thoughts |  |  |  |  |
|  | Follow-up 1 | VUS only | 1.95 | 1.77, 2.14 |
|  | Follow-up 2 | VUS only | 1.86 | 1.66, 2.06 |
|  | Follow-up 1 | P/LP only | 2.19 | 1.75, 2.62 |
|  | Follow-up 2 | P/LP only | 2.27 | 1.87, 2.66 |
|  | Follow-up 1 | P/LP + VUS | 2.07 | 1.62, 2.53 |
|  | Follow-up 2 | P/LP + VUS | 2.13 | 1.66, 2.59 |
| Certainty |  |  |  |  |
|  | Follow-up 1 | VUS only | 3.33 | 3.16, 3.51 |
|  | Follow-up 2 | VUS only | 3.65 | 3.45, 3.84 |
|  | Follow-up 1 | P/LP only | 3.65 | 3.24, 4.07 |
|  | Follow-up 2 | P/LP only | 3.81 | 3.43, 4.19 |
|  | Follow-up 1 | P/LP + VUS | 3.82 | 3.38, 4.25 |
|  | Follow-up 2 | P/LP + VUS | 4.16 | 3.72, 4.61 |
| Self-Efficacy |  |  |  |  |
|  | Follow-up 1 | VUS only | 3.41 | 3.22, 3.60 |
|  | Follow-up 2 | VUS only | 3.76 | 3.56, 3.97 |
|  | Follow-up 1 | P/LP only | 3.67 | 3.22, 4.12 |
|  | Follow-up 2 | P/LP only | 3.61 | 3.21, 4.02 |
|  | Follow-up 1 | P/LP + VUS | 3.22 | 2.76, 3.68 |
|  | Follow-up 2 | P/LP + VUS | 3.89 | 3.41, 4.37 |

Table S3. Sensitivity analysis results from nested mixed-effect model accounting for clustering of parents within child

| **Outcome** | **Estimate** | **95% CI** | **P** |
| --- | --- | --- | --- |
| **Distress** |  |  |  |
| Timepoint | -0.68 | -2.61, 1.26 | 0.492 |
| P/LP result | 5.96 | 1.25, 10.68 | **0.013** |
| VUS result | 1.30 | -1.17, 3.77 | 0.301 |
| P/LP + VUS result | 8.55 | 3.58, 13.52 | **0.001** |
| Days since result disclosure | 0.00 | -0.004, 0.006 | 0.759 |
| Timepoint * P/LP result | 0.80 | -3.37, 4.96 | 0.707 |
| Timepoint * VUS result | 0.13 | -2.09, 2.35 | 0.910 |
| Timepoint * P/LP + VUS result | -4.12 | -8.47, 0.23 | 0.063 |
| **Positive Feelings** |  |  |  |
| Timepoint | -0.29 | -1.29, 0.71 | 0.564 |
| P/LP result | 2.76 | 0.58, 4.94 | **0.013** |
| VUS result | 1.48 | 0.31, 2.64 | **0.013** |
| P/LP + VUS result | 3.73 | 1.38, 6.09 | **0.002** |
| Days since result disclosure | 0.00 | -0.0004, 0.004 | 0.111 |
| Timepoint * P/LP result | 1.01 | -1.15, 3.18 | 0.358 |
| Timepoint * VUS result | -0.17 | -1.36, 1.01 | 0.772 |
| Timepoint * P/LP + VUS result | 0.39 | -1.90, 2.69 | 0.735 |
| **Intrusive Thoughts** |  |  |  |
| Timepoint | -0.10 | -0.35, 0.14 | 0.404 |
| P/LP result | 0.19 | -0.28, 0.66 | 0.432 |
| P/LP + VUS result | 0.13 | -0.36, 0.63 | 0.595 |
| Days since result disclosure | 0.00 | -0.0006, 0.0006 | 0.970 |
| Timepoint * P/LP result | 0.24 | -0.26, 0.75 | 0.340 |
| Timepoint * P/LP + VUS result | 0.17 | -0.38, 0.72 | 0.536 |
| **Certainty** |  |  |  |
| Timepoint | 0.31 | 0.09, 0.54 | **0.007** |
| P/LP result | 0.32 | -0.13, 0.77 | 0.159 |
| P/LP + VUS result | 0.50 | 0.02, 0.98 | **0.040** |
| Days since result disclosure | -0.001 | -0.002, -0.0003 | **0.006** |
| Timepoint * P/LP result | -0.14 | -0.59, 0.30 | 0.527 |
| Timepoint * P/LP + VUS result | 0.03 | -0.46, 0.52 | 0.896 |
| **Self-Efficacy** |  |  |  |
| Timepoint | 0.36 | 0.08, 0.63 | **0.011** |
| P/LP result | 0.26 | -0.21, 0.73 | 0.276 |
| P/LP + VUS result | -0.19 | -0.67, 0.30 | 0.448 |
| Days since result disclosure | -0.001 | -0.002, -0.0004 | **0.001** |
| Timepoint * P/LP result | -0.41 | -1.01, 0.18 | 0.173 |
| Timepoint * P/LP + VUS result | 0.31 | -0.34, 0.96 | 0.343 |
